# Changepoint detection in base-resolution methylome data reveals a robust signature of methylated domain landscape

**DOI:** 10.1186/s12864-015-1809-5

**Published:** 2015-08-12

**Authors:** Takao Yokoyama, Fumihito Miura, Hiromitsu Araki, Kohji Okamura, Takashi Ito

**Affiliations:** Department of Computational Biology, Graduate School of Frontier Sciences, University of Tokyo, 5-1-5 Kashiwanoha, Kashiwa 277-8561, Japan; Department of Biochemistry, Kyushu University Graduate School of Medical Sciences, 3-1-1 Maidashi, Higashi-ku, Fukuoka 812-8582, Japan; Core Research for Evolutional Science and Technology (CREST), Japan Science and Technology Agency (JST), 3-1-1 Maidashi, Higashi-ku, Fukuoka 812-8582, Japan; Department of Systems Biomedicine, National Research Institute for Child Health and Development, National Center for Child Health and Development, 2-10-1 Okura, Setagaya-ku, Tokyo 157-8535, Japan

## Abstract

**Background:**

Base-resolution methylome data generated by whole-genome bisulfite sequencing (WGBS) is often used to segment the genome into domains with distinct methylation levels. However, most segmentation methods include many parameters to be carefully tuned and/or fail to exploit the unsurpassed resolution of the data. Furthermore, there is no simple method that displays the composition of the domains to grasp global trends in each methylome.

**Results:**

We propose to use changepoint detection for domain demarcation based on base-resolution methylome data. While the proposed method segments the methylome in a largely comparable manner to conventional approaches, it has only a single parameter to be tuned. Furthermore, it fully exploits the base-resolution of the data to enable simultaneous detection of methylation changes in even contrasting size ranges, such as focal hypermethylation and global hypomethylation in cancer methylomes. We also propose a simple plot termed methylated domain landscape (MDL) that globally displays the size, the methylation level and the number of the domains thus defined, thereby enabling one to intuitively grasp trends in each methylome. Since the pattern of MDL often reflects cell lineages and is largely unaffected by data size, it can serve as a novel signature of methylome.

**Conclusions:**

Changepoint detection in base-resolution methylome data followed by MDL plotting provides a novel method for methylome characterization and will facilitate global comparison among various WGBS data differing in size and even species origin.

**Electronic supplementary material:**

The online version of this article (doi:10.1186/s12864-015-1809-5) contains supplementary material, which is available to authorized users.

## Background

Cytosine methylated at its C5 position or 5-methylcytosine (5mC) in genomic DNA plays pivotal roles in a wide variety of biological processes. In mammalian cells, 5mC residues principally occur in the context of 5′-CG-3′ dinucleotides or CpG sites, and those at promoters are generally regarded as an important epigenetic mark that leads to gene silencing. Since some transcription factors fail to recognize the methylated versions of their cognate binding sequences, it is formally possible that the cell uses DNA methylation in a sharply focused manner by targeting at a particular CpG site to regulate gene expression. However, most 5mC residues seem to be controlled not independently but more or less coordinately with their neighbours, leading to generate a domain wherein all CpG sites show a largely similar methylation level.

Recent advent of next-generation sequencing technologies has enabled whole-genome bisulfite sequencing (WGBS) to provide highly quantitative methylation data for every cytosine residue in the genome [1, 2]. The base-resolution methylome data is used to segment the methylome into domains with distinct methylation levels. It had been thought that vertebrate genomes are globally methylated to a high level but punctuated with small unmethylated regions, which often include promoters, enhancers, CpG islands and so on [3]. WGBS has not only confirmed this notion but led to finer definitions of methylated domains, revealing those that may be characteristic to developmental stages, tissues or disease states. For instance, the first human WGBS revealed partially methylated domains (PMDs) in fibroblast IMR90 but not in embryonic stem cell (ESC) H1 [4]. On the other hand, a WGBS study on mouse ESC led to propose unmethylated regions (UMRs), low-methylated regions (LMRs) and fully methylated regions (FMRs) [5], whereas another one on four ESC-derived cell types, which reflect early developmental lineages, led to a proposal of a domain termed DNA methylation valley (DMV) [6].

While such methylated domains are often intuitively obvious upon genome browsing, their boundaries should be rationally defined. However, the methods used thus far require substantial optimization steps and have various limitations in domain identification. For instance, the study that proposed PMDs used a sliding window-based approach [4]. While this approach is simple and easy, it cannot detect domains smaller than the pre-defined window size, which is often determined in a rather *ad hoc* manner, depending on the purpose of investigations. Furthermore, window-based approaches use only the mean methylation level of CpG sites in each window, thereby failing to fully exploit the base-resolution of WGBS data. The study that proposed UMRs, LMRs and HMRs used hidden Markov model (HMM) to classify individual CpG sites into three DMA methylation states [5]. While the call of HMM is performed at base-resolution, one has to determine the number of methylation states beforehand and the criteria to define domains based on the states of individual CpG sites.

As an alternative approach to define methylated domains, we tested the feasibility of using changepoint detection. Detecting points of change in a serial measurement data is a general problem that is important not only for natural sciences (*e.g.*, climatology, oceanography, *etc*.) but also in many other areas including the analysis of network traffic and finance [7]. Estimating points of change from statistical properties of measurement data is termed as changepoint detection. In the field of genomics, it has been successfully applied to detect chromosomal copy number variations from array comparative genomic hybridization data [8, 9] and, more recently, from next-generation sequencing data [10], proving its excellent performance [11].

While changepoint detection has demonstrated its power in various areas, it suffers from enormous computational burden. It basically uses a cost function *C* to compare the sum of cost between before and after each domain separation. When examining the changes in mean value of measurement, the cost function is defined as:

(1) $$ C\left(k:n\right)={{\displaystyle {\sum}_{i=k}^n\left({X}_i-\mu \right)}}^2 $$

where *k* and *n* are the start and end points of a domain, respectively, and *X*_*i*_ is a measured value at point *i*, whereas *μ* is the mean value of measurements in the domain. To place a changepoint *t* in a measurement of size *N*, one should satisfy the following formula:

(2) $$ C\left(1:N\right)>C\left(1:t\right)+C\left(t+1:N\right)+\beta $$

where *β* is the penalty value to prevent over-segmentation. The optimal changepoint is identified as the *t* that can minimize the value of the right side of formula (2). Since the computational cost to identify a changepoint *t* that separates a measurement into two domains increases linearly with the size of measurement *N*, the calculation is *O(N)*. Although dynamic programing can consider all possible combinations between segments to detect multiple changepoints [12, 13], the calculation has a computational cost of *O(N*^*3*^*)*, which makes its application to large data including WGBS prohibitive. However, a recently proposed algorithm termed Pruned Exact Linear Time (PELT) has enabled efficient calculation to detect multiple changepoints in almost linear computational time [14].

In this study, we applied the multiple changepoint detection procedure of PELT to base-resolution methylome data generated by WGBS to determine the boundaries of methylated domains or domains with distinct methylation levels. We also devised a novel plot termed methylated domain landscape (MDL) to grasp the global composition of the domains thus defined and found that the pattern of the plot can serve as a signature of each methylome.

## Results

### Changepoint detection in base-resolution methylome data

**Fig. 1:**
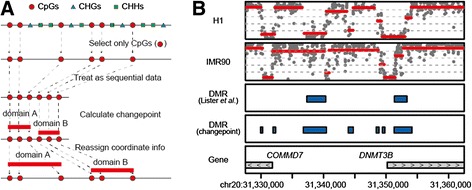
Changepoint detection in base-resolution methylome data. **a** A scheme for application of changepoint detection to base-resolution methylome data. **b** Comparison of methylome data for human ESC H1 and fibroblast IMR90 by Lister *et al.* [4]. A genomic region containing promoters for *COMMD7* and *DNMT3B* genes (chr20, 31,327,500–31,362,500) is shown. Gray dots indicate methylation levels of individual CpG sites that are covered by at least ten reads. Red horizontal bars indicate methylated domains defined by changepoint detection. The vertical level of each bar indicates the mean methylation level of each domain. Blue bars indicate DMRs detected by Lister *et al.* [4] and by changepoint detection

To test whether changepoint detection can define the boundaries of a domain with a distinct methylation level from base-resolution methylome data, we used the WGBS data of human ESC H1 and human lung fibroblast IMR90 [4]. While the ESC methylome includes a substantial number of methylated non-CpG sites, this study focuses on CpG sites, the major targets of mammalian DNA methylation. Since CpG sites do not appear in the genome with a constant interval, we first intended to take the distance between adjacent CpG sites into account to define the domains. However, we could not rationally select any objective model for the purpose. We thus dared to ignore the distance and regarded the CpG methylation data as a simple successive series of measurement data (Fig. 1a). We used the PELT procedure through the R package “changepoint” [14] without any modification to identify boundaries. Following the calculation, we assigned original genomic coordinates to the identified changepoints and displayed each domain by a horizontal line that connects the adjoining changepoints, the vertical level of which indicates the mean methylation level. Judged from their appearance, the domains thus defined were mostly reasonable and of acceptable quality (Fig. 1b). As shown in Fig. 1b, several domains showed different methylation status between the two cell types and were largely coincident with the differentially methylated regions (DMRs) defined by a window-based approach [4]. Since even naïve application of changepoint detection was found to be useful for the analysis of DNA methylation patterns, we decided to pursue this approach more practically by preparing a PELT implementation of our own that was optimized for efficient calculation of genome-scale data in a parallel computational environment.

### Penalty value to control sensitivity in domain demarcation

**Fig. 2:**
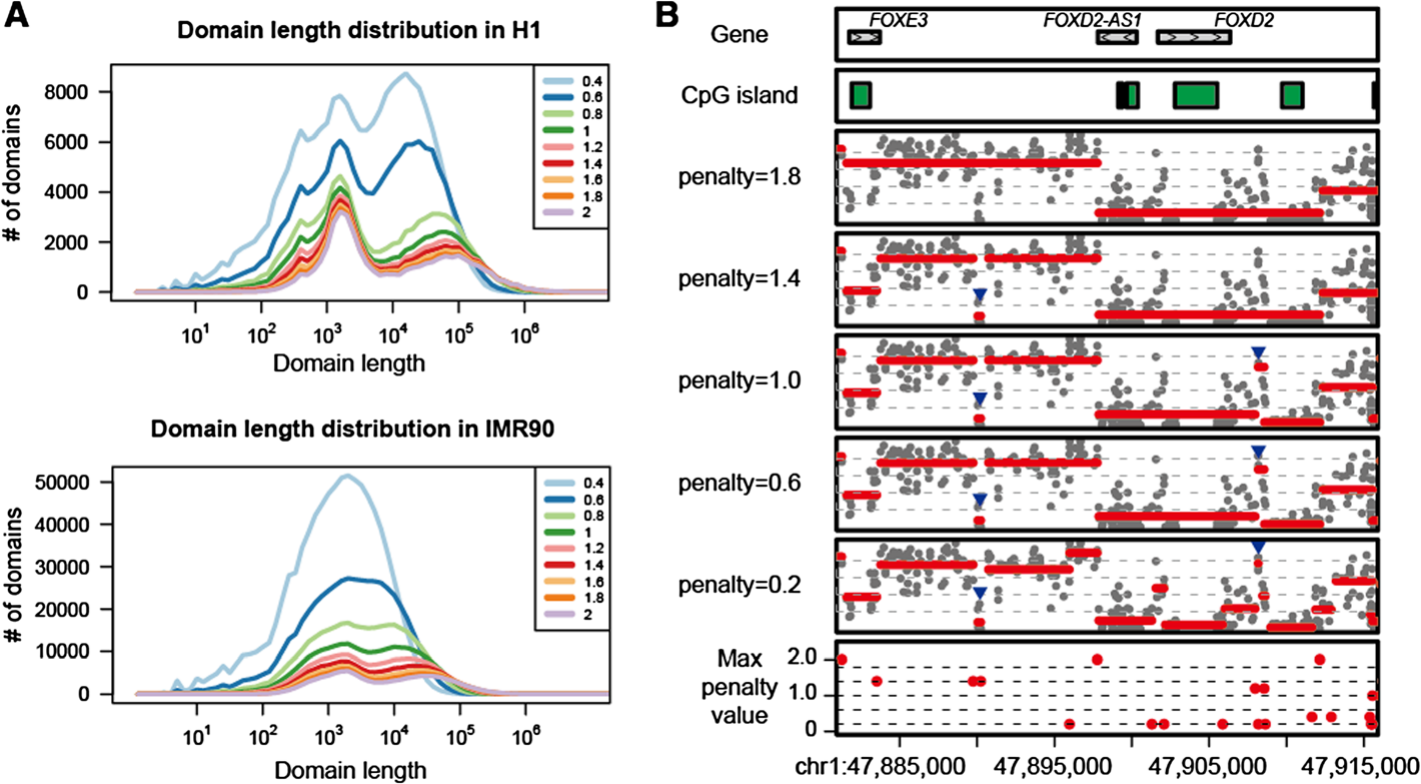
Effects of penalty value on domain demarcation. **a** Effects of penalty value on domain length distribution. CpG sites covered by at least ten reads were used for calculation. **b** Domain demarcation patterns are shown for a genomic region around *FOXD2* gene (chr1, 47,880,913–47,915,913) under five different penalty values. Arrowheads indicate small domains that were missed unless low penalty value were used. The bottom track indicates the maximum penalty value above which each changepoint cannot be detected

To prevent over-segmentation, changepoint detection uses a penalty value for placement of boundaries (see formula (2)). The original implementation of PELT in “changepoint” package [14] makes the penalty value dependent on the size of data to normalize information content. We, however, used a fixed penalty value independently of data size, since the sensitivity of changepoint detection should not differ from one chromosome to another. As expected, the higher the penalty value was, the smaller the number of the detected domains was (Fig. 2a and b). For instance, many small domains of low methylation levels were missed, unless low penalty values were used (Fig. 2b, arrowheads). These domains seem to include such regions around promoters that escape methylation. While detection of such domains is important, too low penalty values result in detection of very tiny domains, most of which contain only a single CpG site (Fig. 2a). Through careful evaluation of the effects of penalty values from a practical point of view, we empirically decided to use a single penalty value of 1.0 throughout this study unless otherwise noted, because it seemed to give, in most cases, a good balance between sensitivity and specificity in domain detection. Note that changepoint detection requests its users to tune only a single parameter, in contrast with window-based approaches, which have, at least, three parameters (*i.e.*, window size, step size and the criteria to merge neighbouring windows) to be optimized depending on the purpose of analysis.

We also assume that the inverse relationship between penalty value and detection sensitivity can serve as a measure of reliability for each boundary. Iterative calculation of boundaries under various penalty values can identify the maximum penalty value above which each boundary cannot be detected anymore. The plot of the maximum penalty value for each boundary would be helpful to interpret domain demarcation (Fig. 2b).

### Visualization and evaluation of changepoint detection-defined domains

**Fig. 3:**
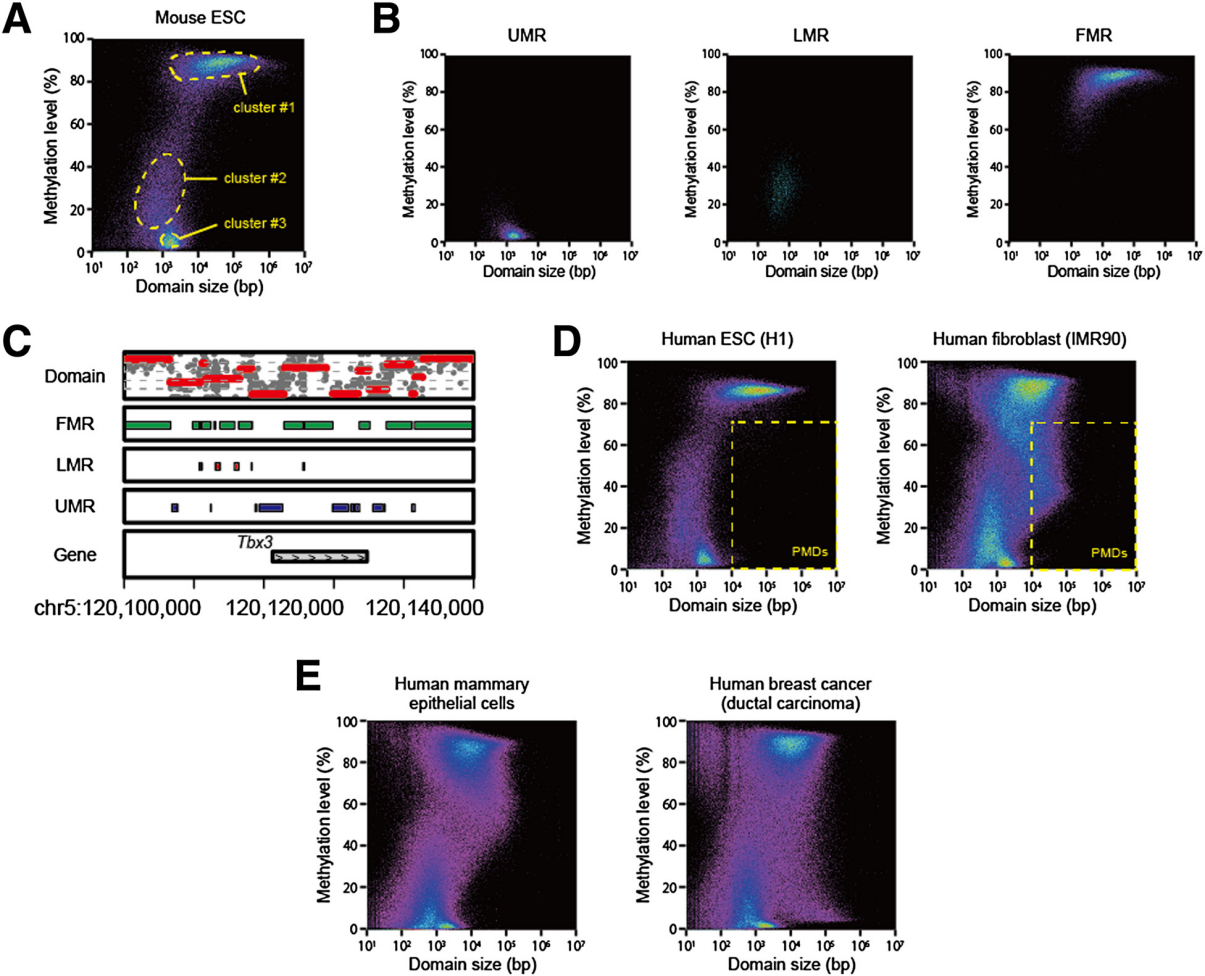
MDL plots for methylated domain composition. **a** MDL plot for mouse ESC [5]. CpG sites covered by at least five reads were used for calculation. Clusters #1 – #3 indicate three major clusters of domains observed in most MDL plots. **b** Distribution of UMRs, LMRs and FMRs on MDL plot. If more than 80 % of a changepoint detection-defined domain overlaps with previously defined UMRs, LMRs or FMRs [5], the domain was selected and displayed in the plots labelled as UMR, LMR or FMR, respectively. **c** Comparison of changepoint detection-defined domains with UMRs, LMRs and FMRs in a genomic region around *Tbx3* gene (chr5, 120,100,000–120,500,000). **d** MDL plots for human ESC H1 and fibroblast IMR90 [4]. Yellow dotted lines demarcate the area that fulfils the criteria used to define PMDs in the original study [4]. **e** MDL plots for human mammary epithelial cells and a low-passage breast cancer cell line [18]

While genome browser enables local visualization of domain demarcation status, it cannot show global trends in methylome segmentation or the composition of domains (*i.e.*, number, size and methylation level of domains). For this purpose, we plotted the size and the mean methylation level of each domain on horizontal and vertical axes, respectively, and used pseudocolor encoding to indicate relative density of the domains. We termed this simple plot as methylated domain landscape (MDL). Examples of MDL were shown for various publicly available WGBS data (Fig. 3 and Additional files 1 and 2). Intriguingly, the domains are not evenly distributed on the plane but rather confined to limited areas. MDL plots generally take a shape that looks like the head and neck of a bird and contains three major clusters of domains. The first cluster is composed of highly methylated domains whose sizes range from 1 kb to several Mb (cluster #1 in Fig. 3a). The second cluster, which is often less prominent than the other two, is composed of short domains ranging from 100 bp to 1.5 kb with low-to-moderate methylation levels (cluster #2 in Fig. 3a). The third cluster is composed of unmethylated domains ranging from 1 kb to 3 kb (cluster #3 in Fig. 3a).

It is intriguing to note that a previous study applied an HMM approach to a mouse ESC data to define three classes of regions termed FMRs, LMRs and UMRs [5]. To test whether these classes correspond to the three clusters observed in MDL plot, we applied the changepoint detection to the same mouse ESC data, extracted such domains that overlap with the FMRs, LMRs or UMRs, and displayed each of them as a separate plot (Fig. 3b and Additional file 3). The changepoint detection-defined domains did not completely coincide with the HMM-defined regions (Fig. 3c). However, the domains overlapping with each class were clearly separated on MDL plot and largely coincident with the three clusters (Fig. 3b and Additional file 3). We also used MethySeekR [15] derived from the HMM-based approach [5] to confirm the overlap between the clusters and the classes (Additional file 3). These results would serve as a proof of concept for the changepoint detection approach. Conversely, we applied MDL plot to the result of HMM approach [5] and observed a pattern largely similar to that by changepoint detection. Curiously, however, the MDL plot of HMM-mediated segmentation revealed an unnatural gap indicating the apparent absence of domains methylated at the level of ~50 % for unknown reason (Additional file 3).

While most MDL plots shared the bird’s head and neck-like shape, some showed substantial variations. For instance, MDL plots were distinct between ESC H1 and fibroblast IMR90 (Fig. 3d), because the latter, but not the former, have many large genomic regions with moderate methylation levels termed PMDs in a previous study [4]. This study defined PMDs as any region that is larger than 10 kb and shows a mean methylation level below 70 %. It was obvious from the MDL plots that IMR90, but not H1, has many domains that fulfil the criteria described above (Fig. 3d and Additional file 4). Indeed, 79 % of the genomic regions in these changepoint detection-defined PMD-like domains overlapped with 75 % of those in the PMDs defined in the original study (Additional file 4). These results indicate that the changepoint detection approach was largely comparable to the window-based approach in terms of detecting PMDs. On the other hand, the MDL plots readily revealed that the IMR90 methylome has many methylated domains that fall short of fulfilling the original PMD criteria but seem not to be rationally separable from PMDs (Fig. 3d). The presence of such PMD-like domains was also obvious in placenta in both human [16] and mouse [17], human mammary epithelial cells and a breast cancer cell line [18] (Additional files 1 and 2). These results may argue a need for redefinition of PMDs.

In this context, it should be noted that the PMD-like domains defined by changepoint detection under a penalty value of 1.0 were generally smaller than the original PMDs defined by a window-based approach: a single large PMD by the original definition was often regarded as a cluster of many smaller PMDs by changepoint detection (Additional file 4). This is because the latter approach was much more sensitive to the presence of tiny unmethylated regions than the former approach. Indeed, changepoint detection under an extreme penalty value ignored such regions, thereby making the domain sizes comparable to those of original PMDs (Additional file 4). Intriguingly, MDL plots suggested that changepoint detection can invariably detect a sizable fraction of UMRs, but not LMRs, even under such conditions (Additional file 4). This trend is likely attributable to the contrasting CpG density between UMRs and LMRs [5]. Demarcation of UMRs, which are enriched for CpG’s [5], has significant impacts on the cost function *C* and hence is often allowed even under high penalty values. By contrast, demarcation of LMRs, which are notably CpG-poor [5], has only a limited contribution to the improvement of the cost function *C* and hence is barely allowed under a high penalty value.

We also analysed the WGBS data for mesendoderm, neural progenitor cells, trophoblast-like cells and mesenchymal stem cells, all of which were differentiated from human ESC H1 [6]. These datasets led to the proposal DMV, which was defined as any genomic region larger than 5 kb with a mean methylation level below 15 % [6]. MDL plots indicated the presence of domains that fulfil the criteria (Additional file 5). Consistent with the original study [5], these domains were minorities but found in all of the four cell types. Intriguingly, 66–90 % of the genomic regions in these apparently DMV-like domains overlapped with those in the DMVs defined in the original study (Additional file 5). On the other hand, the former regions failed to cover 38–58 % of the latter regions (Additional file 5). This is because changepoint detection under a penalty value of 1.0 often divided a single DMV defined in the original study into two or more UMR-like domains. As the original DMVs were defined using a sliding window-based approach with a step size of 1,000 bp [6], their boundaries were inevitably blurred and their sizes were likely exaggerated. Notably, the sub-boundary portions of each DMV defined by the original study often showed higher methylation levels than its central portion (Additional file 5). Changepoint detection sensitively recognized such portions as distinct domains to provide intuitively more natural boundaries (Additional file 5). Consequently, changepoint detection-defined DMV-like domains were, in most cases, smaller than original DMVs: some of the former failed to cover substantial portions of the latter, others were not recognized as DMV-like domains anymore because they were smaller than 5 kb (Additional file 5).

It is well known that carcinogenesis often associates two contrasting changes in the methylome, namely global hypomethylation and focal hypermethylation, which have been implicated in chromosome instability and epigenetic suppression of tumor suppressor genes, respectively. We analysed the WGBS data of cultured primary human mammary epithelial cells and a low-passage breast cancer cell line [18]. Comparison between the MDL plots of these cells indicated the emergence of large hypomethylation domains and small hypermethylated domains upon carcinogenesis (Fig. 3e). It should be noted that simultaneous detection of changes in contrasting size ranges is demanding for conventional approaches using a single pre-defined window size, whereas it is readily achievable for the changepoint detection approach.

Taken together, changepoint detection can well demarcate the domains in a largely comparable manner with conventional approaches, and the MDL plot is useful for global visualization of the domains thus defined to reveal the global trends in each methylome.

### MDL plot as a signature of methylome

The observations described above prompted us to pursue a possible use of MDL plot as a unique signature of each methylome. Besides the presence of PMDs, there are substantial variations in MDL plots. For instance, ESC and ESC-derived lineages have less domains than hematopoietic cells (Additional file 6). Among the ESC-derived cell lineages [6], mesendoderm, neural progenitor cells and trophoblast-like cells showed a flat head with a sharp beak, whereas mesenchymal stem cells have a rather round head with a dull beak (Additional file 5). The former and the latter would thus have low and high variance in methylation levels of the highly methylated domains (*i.e.*, cluster #1 or FMRs), respectively. Cells of hematopoietic lineage often have a round head with a dull beak, similarly to mesenchymal stem cells (Additional file 1) [19]. Intriguingly, disruption of *Dnmt3a* gene significantly affected the pattern in hematopoietic stem cells (Additional file 1) [20].

**Fig. 4:**
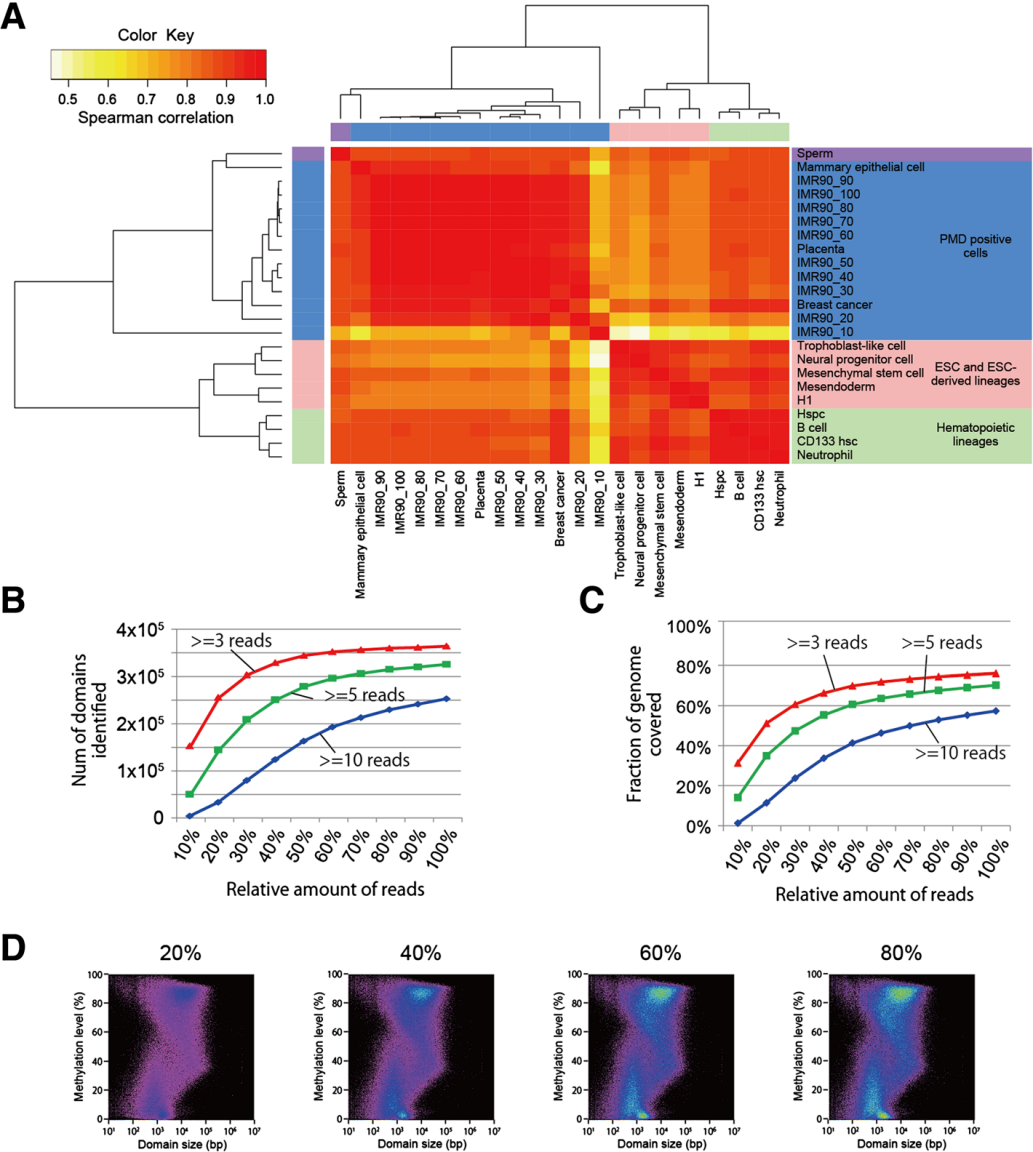
MDL plots as a signature of methylome. **a** Unsupervised clustering of MDL plots. WGBS data for human fibroblast IMR90 and ESC H1 [4], ESC-derived lineages including mesendoderm, trophoblast-like cell, neural progenitor cell and mesenchymal stem cell [6], cultured mammary epithelial cells and a low-passage breast cancer cell line [18], hematopoietic lineage cells including hematopoietic stem/progenitor cell (Hspc), CD133-positive hematopoietic stem cell (CD133 hsc), neutrophil and B-cell [19], placenta [16] and sperm [24] were subjected to changepoint detection followed by MDL plotting. We also included variously down-sized IMR90 data (see below). CpG sites covered by at least five reads were used for calculation. We performed Ward’s clustering using the Spearman’s rank-order correlation coefficient (see Methods). The heatmap illustrates the relationships across cell types based on MDL plots. The graded colors from red to white at the top left represent from similar to dissimilar in terms of the Spearman’s rank-order correlation coefficient between samples. **b** Effects of data size on the number of domains. We segmented the methylomes using the ten variously downsized IMR90 data under three different thresholds or minimal depth to select CpG sites for calculation of methylation level (*i.e.*, ≥3, ≥5 or ≥10 reads). **c** Effects of data size on genomic coverage. **d** Effects of data size on MDL plot of IMR90 cells. The percentile on each plot indicates the fraction of data used for the analysis. CpG sites covered by at least three reads were used for calculation

To objectively evaluate such apparent similarity, we divided each MDL plot into 400 (= 20 × 20) pixels, used the number of domains in each pixel to calculate the Spearman’s rank-order correlation coefficient and performed Ward’s clustering of various methylomes (Fig. 4a and Additional file 6). As expected, the methylomes with PMDs including IMR90, placenta, mammary epithelial cells and a breast cancer cell line were clustered together. Intriguingly, the methylomes of ESC and ESC-derived lineages formed a single cluster, and those of hematopoietic lineages formed another cluster. These results suggested that the extent of methylome segmentation can be unique to cell lineage and hence serve as a novel signature of each methylome. The result of MDL-based clustering was largely consistent with those of conventional clustering based on the methylation levels of either individual CpG sites or sliding windows (Additional file 6). However, the latter approach placed mesenchymal stem cells in the cluster of PMD-positive cells (Additional file 6). MDL-based clustering can be thus more sensitive, at least, to some types of change than conventional clustering, thereby serving as a novel approach complementary to conventional ones in methylome data analysis.

We next intended to examine the robustness of MDL plot against data size, because the number of reads differ substantially among various WGBS data. In principle, the deeper one sequences the methylome, the more accurately one can estimate methylation levels. Accordingly, a threshold for read depth is usually set to select CpG residues used for the calculation of methylation levels, while discarding the others from subsequent analysis. In other words, shallow sequencing depth tends to lose both accuracy and coverage. We thus examined the effects of read depth on domain demarcation by changepoint detection. For this purpose, we used the WGBS data for IMR90 [4], because it covers the human genome by more than 40-fold, when combining the two biological replicate data. We prepared ten IMR90 datasets whose sizes were 10–100 % of that of the full data, and used each of them to calculate domain boundaries under three different threshold values (*i.e.*, minimal read depths) to calculate methylation levels. As expected, the number of domains and the genomic coverage were dependent on the amount of reads used for the calculation (Fig. 4b and c). Higher thresholds requested more reads to detect the same number of domains or to achieve the same genomic coverage (Fig. 4b and c). Total number of domains increased not proportionally to the amount of reads but rather approaching to a plateau (Fig. 4b). The overall appearance of MDL plot was notably unaffected even upon substantial reduction of data especially under low threshold values, although too extensive deletion of data (*e.g.*, 90 % deletion) resulted in an increase in domain size and subsequent right-ward shift of the plot (Fig. 4d and Additional file 7). The result of clustering also supported the substantial robustness of MDL plots (Fig. 4a).

These results indicated that changepoint detection followed by MDL plotting enables one to grasp the trends in methylome largely independently of data size, that is, even from rather shallow data. We thus expect MDL plot can serve as a robust signature of each methylome to facilitate comparison among WGBS data substantially differing in their sizes.

## Discussion

This work proposes an application of changepoint detection to demarcate domains in the methylome based on base-resolution data obtained by WGBS (Fig. 1). Thanks to the efficient algorithm termed PELT [14], human WGBS data can be analysed within 20 min with a single thread of processor: our implementation of changepoint detection was competitive enough or even superior to other approaches for methylome segmentation (Additional file 8). Another advantage of this method would be its simplicity in optimization, since it requires only a single parameter termed “penalty” to be tuned for sensitivity control (Fig. 2).

Despite the lightness of calculation and the simplicity in parameter optimization, changepoint detection can well segment the methylome in a largely comparable manner with previous approaches, detecting both ubiquitous and cell type-specific methylated domains (Fig. 3). Of note, even a single penalty value of 1.0 can substantially, albeit not optimally, cover various methylome characteristics, each of which was originally defined using a distinct set of optimized parameters. While the penalty value of 1.0 is a good point to start with, we also recommend users to increase or decrease the penalty value depending on the size and CpG-density of the domains on which they intend to put emphasis (see below).

Changepoint detection uses every CpG dinucleotide for calculation to fully exploit the base-resolution. This is an obvious advantage of changepoint detection over window-based approach, because the former has much higher spatial resolution than the latter, resolution of which is inevitably limited by the sizes of window and step. Accordingly, the former often provides intuitively more natural boundaries than the latter (Additional file 5). Furthermore, changepoint detection can cover changes of any size, in sharp contrast with window-based approach, which is insensitive to changes whose sizes are smaller than the window size. It can thus simultaneously detect such changes that occur even in contrasting size ranges, including global hypermethylation and focal hypomethylation in cancer methylomes (Fig. 3e). On the other hand, changepoint detection tends to induce over-segmentation of large features including PMDs and DMVs, both of which were originally defined by window-based approaches. Although it remains elusive and/or depends on the purpose of studies whether or not one should stick to the original definitions, one can simply use higher penalty values to obtain results largely comparable to those in the original studies (Additional file 4).

HMM-based segmentation represents another useful approach that fully exploits the base-resolution of WGBS data [5]. However, to use this approach, one has to determine the number of states as well as the process to define a domain based on the states of CpG sites, both of which cannot be rationally deduced and would require substantial optimization through trials and errors. By contrast, changepoint detection requests its users to tune only the penalty value but not any criteria for subsequent domain definition. Intriguingly, these two approaches share a problem of PMD over-segmentation. To overcome this issue, MethylSeekR included an HMM-based masking of PMDs prior to identification of LMRs and UMRs [15]. While a similar two-step approach may be plausible for changepoint detection, the effect of over-segmentation can be readily mitigated by simply increasing the penalty value (Additional file 4). We thus note that changepoint detection can be flexibly adapted to various modifications, since it has only a single parameter to be tuned.

Changepoint detection would be also useful for the detection of DMRs. As shown in Fig. 1b, comparison of calculated domains between two methylome data readily led to the identification of previously described DMRs: DMR can be identified either as a domain sharing the same boundaries but with different methylation levels between the two data or as any shift of boundaries.

Another limitation of changepoint detection that we are currently aware of is that it cannot detect a region with a gradually increasing or decreasing methylation level, since the algorithm is based on a model postulating that a domain is consist of data points with a single mean value with a single dispersion. In principle, a slope in methylation level should be a reflection of underlying cellular heterogeneity or cell-to-cell variation of domain border, presumably caused by a tug-of-war between methylating and demethylating activities. To correctly detect such biologically interesting regions, the method has to employ a different model for domain definition.

Once a methylome is segmented into distinct domains, their overall composition or their number, sizes and methylation levels can serve as a signature of the methylome. To grasp the domain composition intuitively, we devised a representation termed MDL that plots the size and methylation level on the X- and Y-axes, respectively. This simple plot reveals three basic clusters of domains, which largely correspond to the FMRs, LMRs and UMRs defined using an HMM approach (Fig. 3 and Additional file 3). The MDL plot is also useful to examine PMDs (Additional file 4). The presence of PMDs has been indicated in the literature by either browser screenshots of typical PMDs or a scatter plot wherein X- and Y-coordinates of each dot indicate methylation levels of a window in the two samples to be compared [21]. While this plot is useful to compare two methylomes and can indicate the presence of hypomethylated windows, it cannot tell whether or not such windows are continuous or clustered in the genome to constitute PMDs. By contrast, MDL plot explicitly indicates the presence of PMDs as well as their global status, since it displays both the methylation levels and the sizes of each methylated domain. In addition, MDL plot displays domain sizes in a logarithmic scale, it can cover a wide range of domain size. Consequently, comparison of MDL plots between normal mammary epithelial cells and breast cancer cells highlighted the emergence of small hypermethylated domains and large hypomethylated domains in the latter (Fig. 3e).

Intriguingly, the MDL plots of related cell types were often placed in close vicinity on the clustering dendrograms (Fig. 4a). These results may indicate an intriguing possibility of cell lineage-dependent strategy in methylome segmentation, prevalence, underlying mechanisms and biological significance of which remain to be seen in the future studies using a more sophisticated method to evaluate the similarity between MDL plots. In this context, it would be also important to compare methylomes between different species, and MDL plots are readily applicable to such interspecies comparison. For instance, it is obvious from MDL plots that both human and mouse placenta have prominent PMDs. We thus expect that MDL can be a versatile tool for comparative epigenomics.

Furthermore, MDL plot was largely unaffected by the size of WGBS data, retaining its pattern even when the data were substantially deleted (Fig. 4a and d and Additional file 7). As it can extract the global trends of methylome even from rather shallow WGBS data, it would enable direct comparison among WGBS data of various sizes to facilitate their integration.

While we applied changepoint detection to base-resolution methylome data in this study, we also noticed that changepoint detection is useful for analysing ChIP-Seq data to detect peaks of various histone modifications, in particular, broad peaks for suppressive chromatin marks such as trimethylation at the 9th and 27th lysine residues of histone H3 (unpublished observation). Consistently, a similar attempt was recently reported to analyse ChIP-chip data for histone modifications [22].

## Conclusions

Changepoint detection provides a useful method to define methylated domains based on base-resolution methylome data. Global visualization of the domains thus defined on MDL plot reveals a robust signature of each methylome, facilitating comparison of various methylome data.

## Methods

### WGBS data and their mapping

All sequence data used in this study were downloaded from DDBJ Sequence Read Archive and did not include any controlled-access data. Accordingly, this study did not require any ethics approval. WGBS reads were mapped to UCSC human genome 19 (hg19) or mouse genome 9 (mm9), as described previously [23]. However, we used hg18 in comparative analyses on PMDs and DMVs (Additional files 4 and 5), because the original studies on these features were based on hg18 [4, 6].

### Changepoint detection and methylated domain landscape plotting

We processed and visualized the mapped reads using an in-house pipeline. We calculated domains using the R package “changepoint” [14] and an implementation of our own named SimpleChangepointCalculator for the data shown in Fig. 1 and those in Figs. 2, 3 and 4, respectively. Identified domains were visualized using functions of either R or SimpleChangepointCalculator. SimpleChangepointCalculater is available at http://itolab.med.kyushu-u.ac.jp/CPT/.

### Unsupervised clustering of MDL plots

Each MDL plot was divided into 400 (= 20 × 20) pixels. MDLs were then subjected to Ward’s clustering using Spearman’s rank-order correlation coefficient calculated from the number of domains in each pixel.

## Additional files

Additional file 1:
**MDL plots for eight publically available WGBS data.** (PDF 471 kb) Additional file 2:
**MDL plots for WGBS data on 17 mouse adult tissues.** (PDF 850 kb) Additional file 3:
**Comparison between HMM-defined methylated regions and clusters of changepoint-defined domains.** (PDF 278 kb) Additional file 4:
**Comparison of PMDs detected by window-based and changepoint detection approaches.** (PDF 551 kb) Additional file 5:
**Comparison of DMVs detected by window-based and changepoint detection approaches.** (PDF 422 kb) Additional file 6:
**Unsupervised clustering of MDL plots.**(PDF 261 kb) Additional file 7:
**Effects of data size on MDL plots.** (PDF 324 kb) Additional file 8:
**Performance comparison among methylome segmentation tools.** (PDF 192 kb)

## Abbreviations

CD133 hsc: CD133-positive hematopoietic stem cell
DMR: Differentially methylated region
DMV: DNA methylation valley
ESC: Embryonic stem cell
FMR: Fully methylated region
HMM: Hidden Markov model
Hspc: Hematopoietic stem/progenitor cell
LMR: Low-methylated region
5mC: 5-Methylcytosine
MDL: Methylated domain landscape
PELT: Pruned exact linear time
PMD: Partially methylated domain
UMR: Unmethylated region
WGBS: Whole-genome bisulfite sequencing
