# Supplementary material for: Changepoint detection in base-resolution methylome data reveals a robust signature of methylated domain landscape
Source: BMC Genomics. 2015 Aug 12;16(1):594. doi: 10.1186/s12864-015-1809-5 (PMC4534107; doi:10.1186/s12864-015-1809-5)
Supplement: Additional file 1: — MDL plots for eight publically available WGBS data. (PDF 471 kb) [file 12864_2015_1809_MOESM1_ESM.pdf]

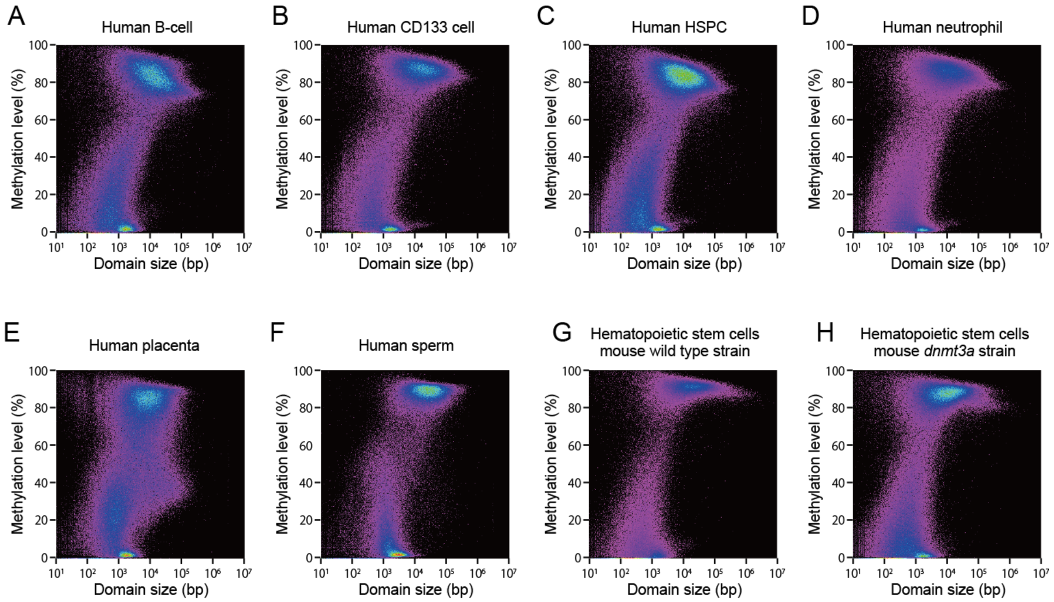

Additional file 1 – MDL plots for eight publically available WGBS data

(A~D) Human hematopoietic lineages [19]. **(E)** Human placenta [16]. **(F)** Human sperm [23]. **(G, H)** Mouse hematopoietic stem cells from wild type and *dnmt3a* KO mice [20].
