# Supplementary material for: Changepoint detection in base-resolution methylome data reveals a robust signature of methylated domain landscape
Source: BMC Genomics. 2015 Aug 12;16(1):594. doi: 10.1186/s12864-015-1809-5 (PMC4534107; doi:10.1186/s12864-015-1809-5)
Supplement: Additional file 2: — MDL plots for WGBS data on 17 mouse adult tissues. (PDF 850 kb) [file 12864_2015_1809_MOESM2_ESM.pdf]

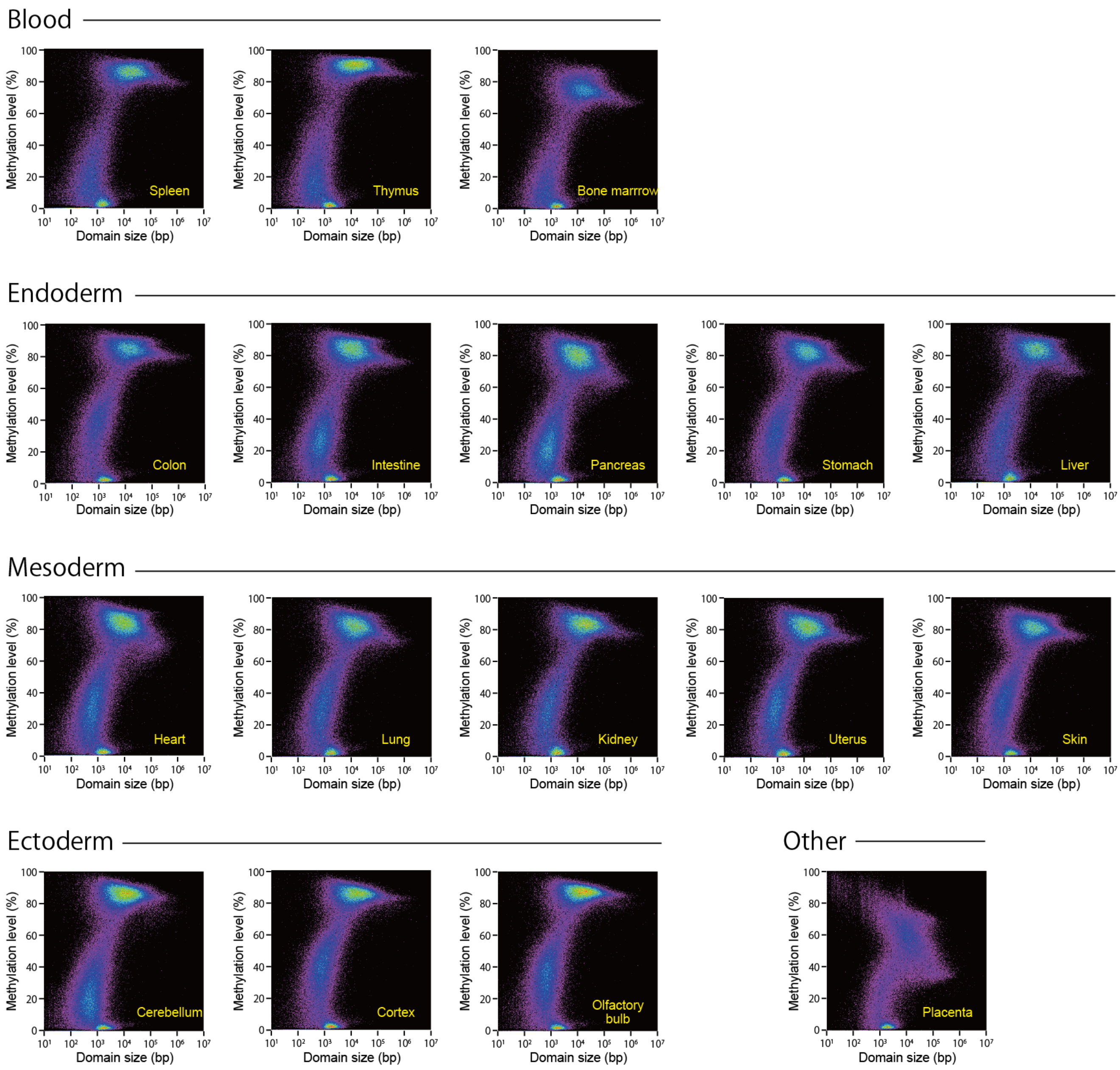

Additional file 2 – MDL plots for WGBS data on 17 mouse adult tissues

Data from [17] were mapped, changepoint-detected and displayed as MDL plots.
