# Supplementary material for: Changepoint detection in base-resolution methylome data reveals a robust signature of methylated domain landscape
Source: BMC Genomics. 2015 Aug 12;16(1):594. doi: 10.1186/s12864-015-1809-5 (PMC4534107; doi:10.1186/s12864-015-1809-5)
Supplement: Additional file 3: — Comparison between HMM-defined methylated regions and clusters of changepoint-defined domains. (PDF 278 kb) [file 12864_2015_1809_MOESM3_ESM.pdf]

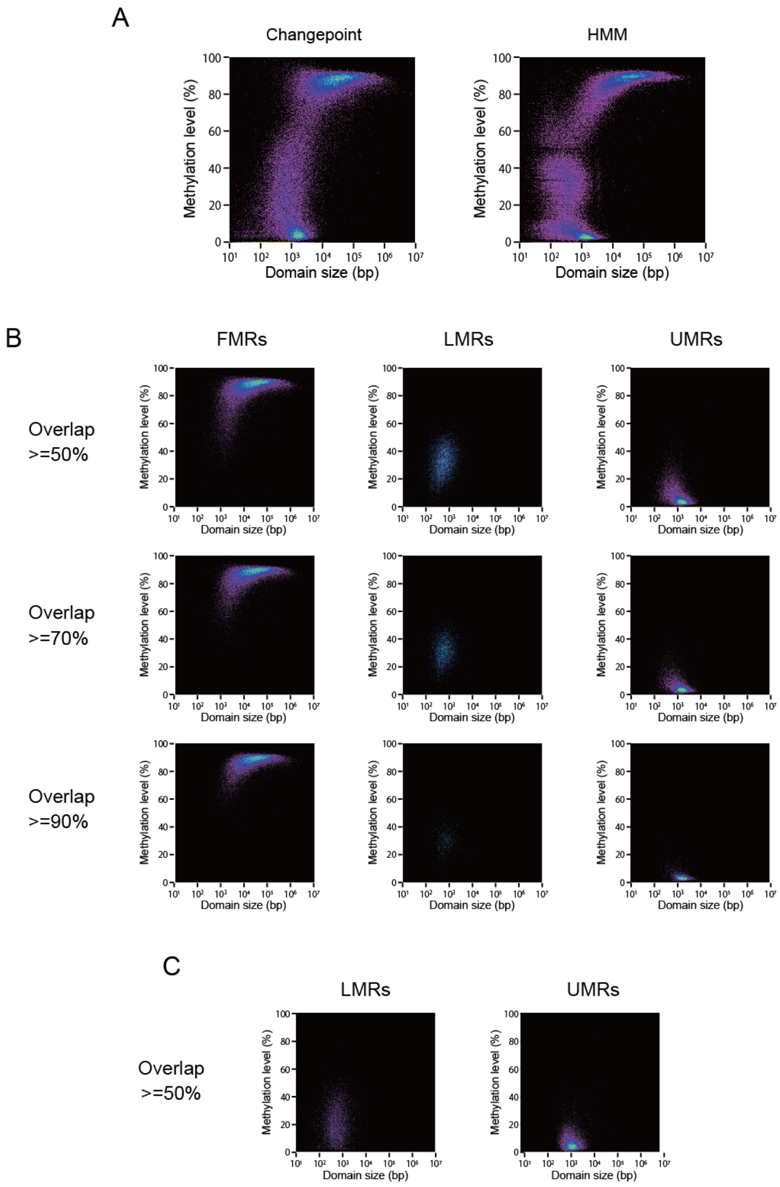

Additional file 3 – Comparison between HMM-defined methylated regions and changepoint-defined domain clusters

(A) MDL plot for mouse ESC (same as Figure 3A). CpG sites covered by at least 5 reads were used for the calculation by changepoint detection (left). The result of [5] was also displayed as an MDL plot (right). **(B)** MDL plots for domains that overlap with FMRs, LMRs and UMRs. If a changepoint detection-defined domain overlaps with FMRs, LMRs or UMRs defined in [5], lengths are calculated for their intersection and union. If the length of the intersection exceeds 50, 70 or 90% of that of the union, then the domain was displayed on the MDL plots accordingly. **(C)** MDL plots for domains that overlap with LMRs and UMRs calculated using MethylSeekR [15]. Domains with the intersection exceeding 50% of the union were displayed.
