# Supplementary material for: Changepoint detection in base-resolution methylome data reveals a robust signature of methylated domain landscape
Source: BMC Genomics. 2015 Aug 12;16(1):594. doi: 10.1186/s12864-015-1809-5 (PMC4534107; doi:10.1186/s12864-015-1809-5)
Supplement: Additional file 4: — Comparison of PMDs detected by window-based and changepoint detection approaches. (PDF 551 kb) [file 12864_2015_1809_MOESM4_ESM.pdf]

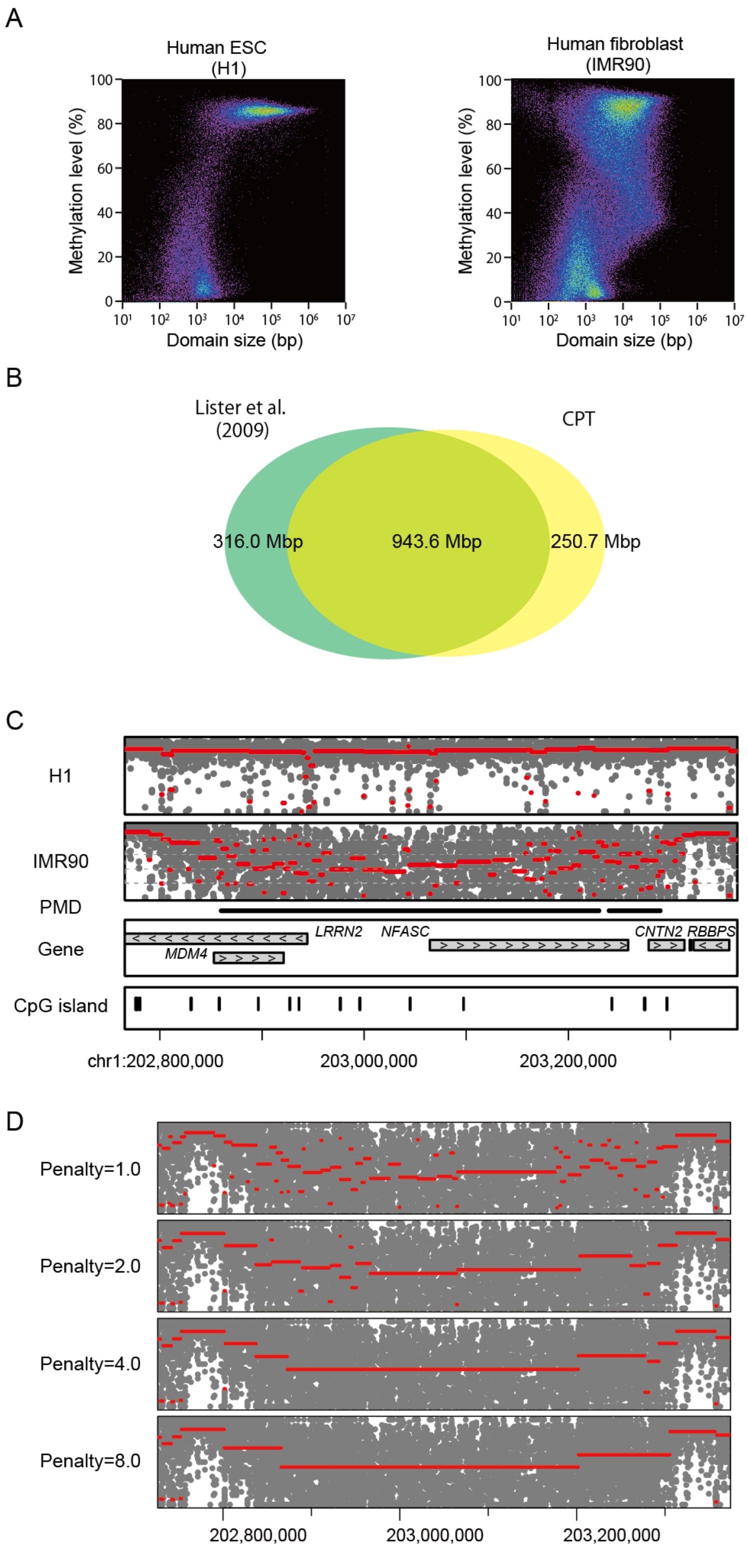
A
Human ESC
(H1)
Human fibroblast
(IMR90)
Methylation level (%)
100
80
60
40
20
0
10^1 10^2 10^3 10^4 10^5 10^6 10^7
Domain size (bp)
B
Lister et al.
(2009)
CPT
316.0 Mbp
943.6 Mbp
250.7 Mbp
C
H1
IMR90
PMD
Gene
CpG island
LRRN2
NFASC
CNTN2
RBBPS
MDM4
chr1:202,800,000
203,000,000
203,200,000
D
Penalty=1.0
Penalty=2.0
Penalty=4.0
Penalty=8.0
202,800,000
203,000,000
203,200,000

E

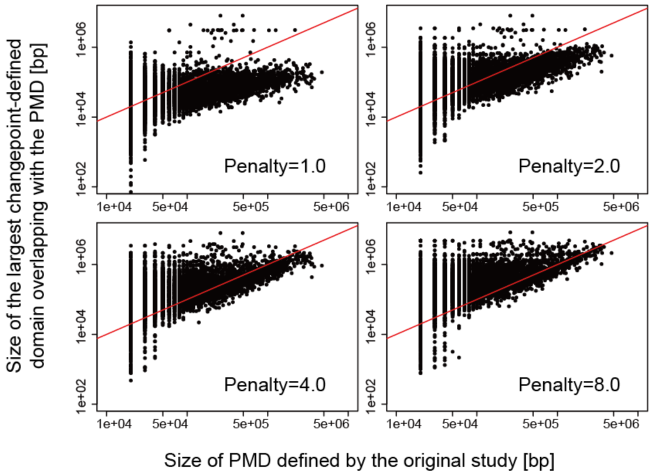

F

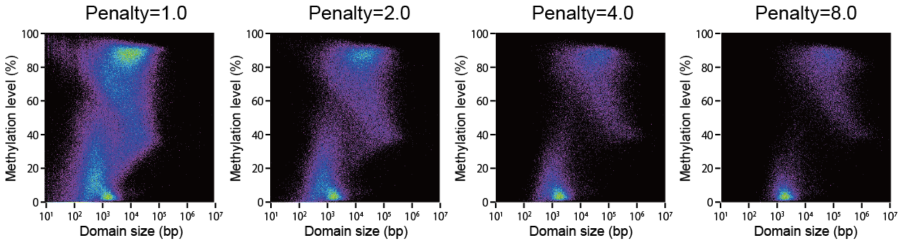

Additional file 4 – Comparison of PMDs detected by window-based and changepoint detection approaches

(A) MDL plots for human ESC H1 and fibroblast IMR90 (same as Figure 3D). **(B)** Venn diagram for the overlap in size among the PMDs defined by Lister et al. [4] and the changepoint detection-defined domains that satisfy the original criteria of PMDs (*i.e.*, larger than 10 kb in size and less than 70% for mean methylation level). **(C)** An example of discrepancy in PMD detection by the two approaches. While the original study [4] identified two PMDs in this region, changepoint detection defined many short domains with variable levels of partial methylation, some of which were smaller than 10 kb. Top two tracks indicate methylation levels (gray dots) and changepoint detection-defined domains (red horizontal bars) for H1 and IMR90. The PMD track displays PMDs defined in [4] as black horizontal bars. Genes and CpG islands are also shown. **(D)** Effects of penalty value on PMD segmentation exemplified by the same locus as shown in (C). **(E)** Effects of penalty value on changepoint-defined PMD-like domains. The size of the largest changepoint-defined domain overlapping with an original PMD was plotted against the size of the original PMD. If the changepoint-defined and the original PMDs share the same size, then the dots should be on the diagonal indicated as a red line. The fractions of PMDs under the diagonal were 67%, 43%, 29% and 21% under the penalty values of 1, 2, 4, and 8, respectively. **(F)** Effect of penalty value on MDL plot of IMR90 cells [4].
