# Supplementary material for: Changepoint detection in base-resolution methylome data reveals a robust signature of methylated domain landscape
Source: BMC Genomics. 2015 Aug 12;16(1):594. doi: 10.1186/s12864-015-1809-5 (PMC4534107; doi:10.1186/s12864-015-1809-5)
Supplement: Additional file 5: — Comparison of DMVs detected by window-based and changepoint detection approaches. (PDF 422 kb) [file 12864_2015_1809_MOESM5_ESM.pdf]

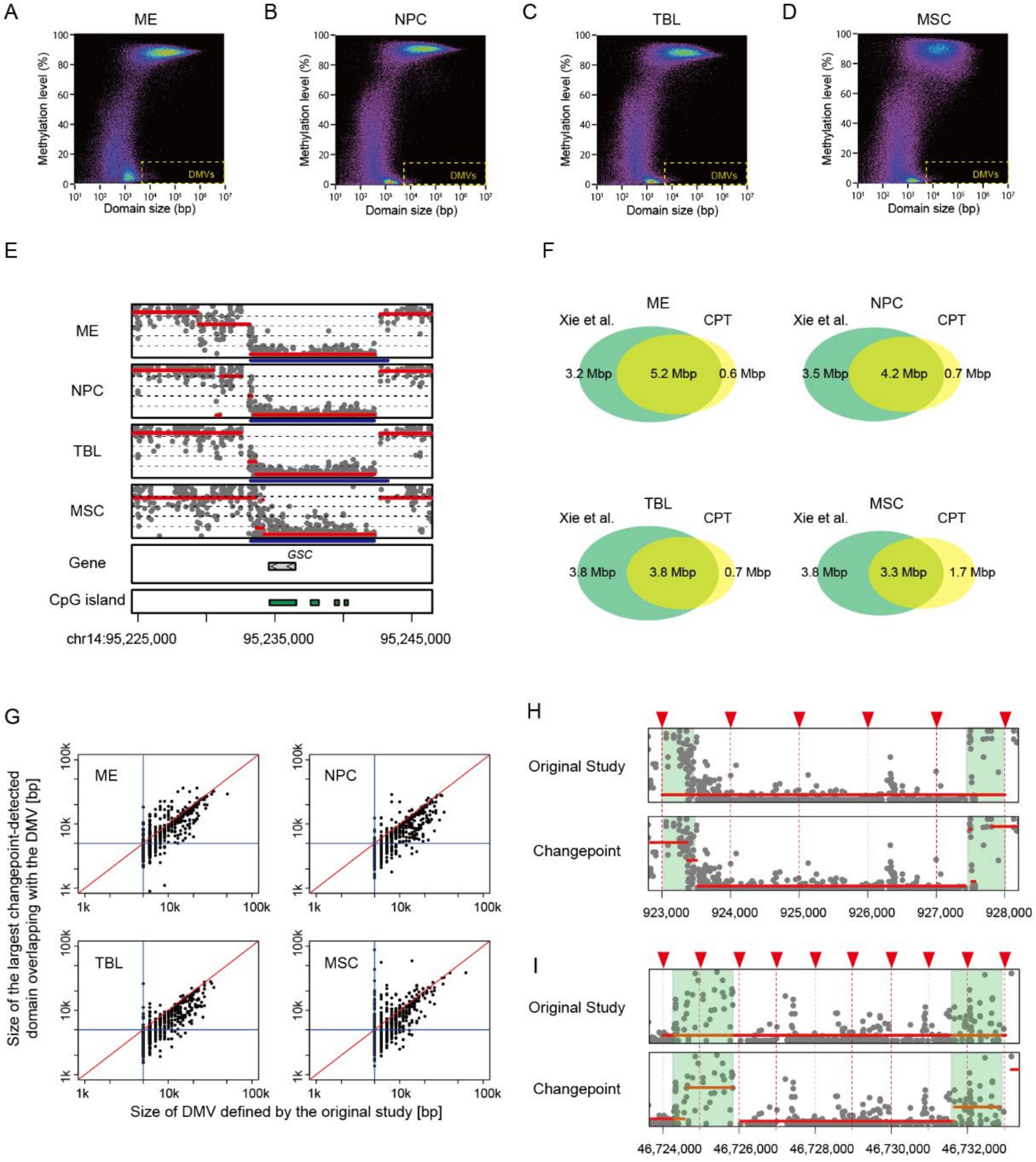

Additional file 5 – Comparison of DMVs detected by window-based and changepoint detection approaches

(A~D) MDL plots for mesendoderm (ME), neural progenitor cell (NPC), trophoblast stem cell (TBL) and mesenchymal stem cell (MSC). **(E)** Example of DMV defined in [6] (blue bars) and changepoint detection-defined domains (red bars) in the four cell types. **(F)** Venn diagram for the overlap in size between the DMVs defined in [6] (green circles labeled as Xie et al.) and the changepoint detection-defined domains whose sizes and methylation levels are ≥5 kb long and

≤15% (yellow circles labeled as CPT), respectively. **(G)** Comparison of domain size between the original DMVs [6] and the overlapping changepoint-defined domains. The size of the largest changepoint-defined domain that overlaps with an original DMV was plotted against the size of the DMV. Blue lines indicate 5 kb. Red lines represent diagonals. Note that most changepoint-defined domains were smaller than the corresponding DMV and that a substantial fraction of changepoint-defined domains were smaller than 5 kb and hence not regarded as DMV. **(H and I)** DMV and overlapping changepoint-defined domains. Red arrow heads and dashed lines indicate boundaries of the windows to calculate DMVs (step size; 1.0 kb) in the original study [6]. Red horizontal bars indicates typical DMVs in mesenchymal stem cells (H, chr1: 923,001–928,000; I, chr1: 46,724,001–46,733,000). Note that sub-boundary regions (light green) are substantially hypermethylated than the central portions with very low methylation levels: the boundaries of these DMVs were obviously blurred by the window-based approach. By contrast, changepoint detection identified the sub-boundary portions as distinct small domains or merged with the flanking regions, thereby providing intuitively much more natural boundaries and substantially smaller domain sizes than the window-based approach.
