# Supplementary material for: Changepoint detection in base-resolution methylome data reveals a robust signature of methylated domain landscape
Source: BMC Genomics. 2015 Aug 12;16(1):594. doi: 10.1186/s12864-015-1809-5 (PMC4534107; doi:10.1186/s12864-015-1809-5)
Supplement: Additional file 6: — Unsupervised clustering of MDL plots.(PDF 261 kb) [file 12864_2015_1809_MOESM6_ESM.pdf]

A

| Sample name | Cell type | Mean depth | Number of domains (length > 10 bp) |
|---|---|---|---|
| H1 | ESC or ESC-derived | 33.3 | 71,283 |
| Mesenchymal stem cell | ESC or ESC-derived | 55.5 | 170,898 |
| Mesendoderm | ESC or ESC-derived | 31.3 | 99,353 |
| Neural progenitor cell | ESC or ESC-derived | 38.9 | 139,413 |
| Trophoblast-like cell | ESC or ESC-derived | 40.6 | 142,294 |
| IMR90_10 | PMD-positive | 4.1 | 50,383 |
| IMR90_20 | PMD-positive | 8.1 | 144,499 |
| IMR90_30 | PMD-positive | 12.1 | 208,981 |
| IMR90_40 | PMD-positive | 16.2 | 250,546 |
| IMR90_50 | PMD-positive | 20.3 | 278,634 |
| IMR90_60 | PMD-positive | 24.4 | 295,620 |
| IMR90_70 | PMD-positive | 28.4 | 305,316 |
| IMR90_80 | PMD-positive | 32.5 | 313,744 |
| IMR90_90 | PMD-positive | 36.4 | 318,776 |
| IMR90_100 | PMD-positive | 40.5 | 324,456 |
| Placenta | PMD-positive | 9.3 | 308,166 |
| Mammary epithelial cell | PMD-positive | 20.1 | 292,602 |
| Breast cancer | PMD-positive | 31.7 | 316,413 |
| B cell | Hematopoietic | 12.3 | 229,717 |
| CD133 hsc | Hematopoietic | 10.6 | 212,253 |
| Hspc | Hematopoietic | 31.7 | 333,716 |
| Neutrophil | Hematopoietic | 10.0 | 256,623 |
| Sperm | Sperm | 7.3 | 162,496 |

B

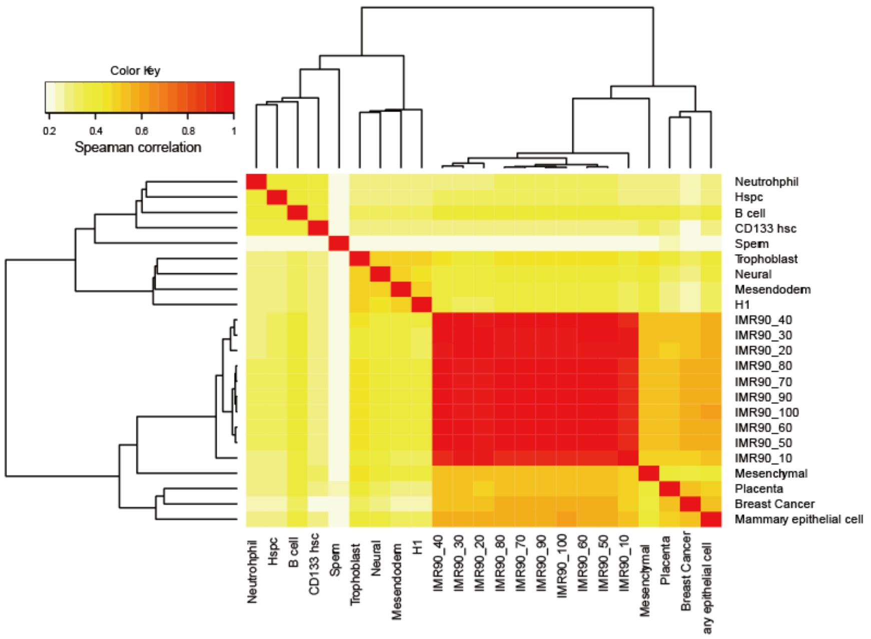


C

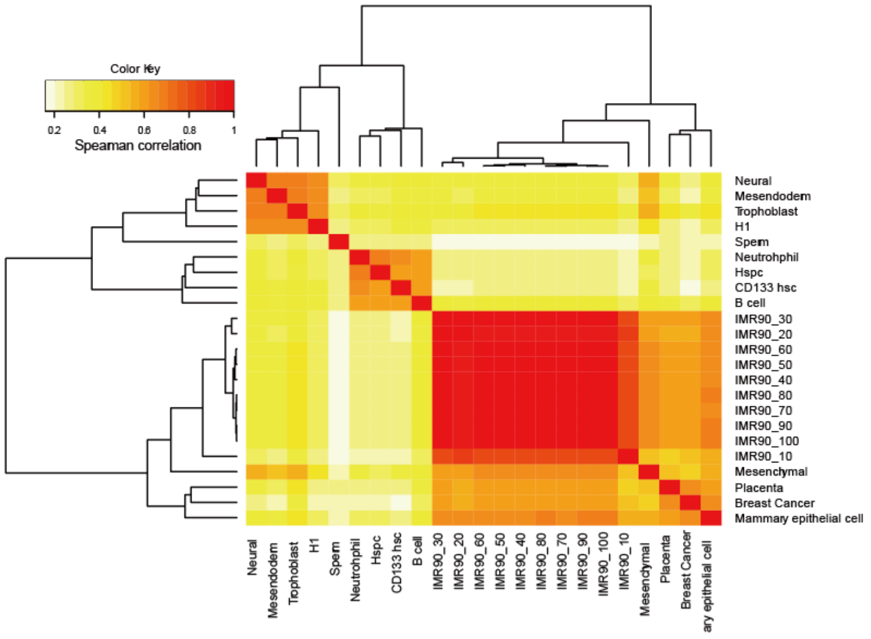


## Additional file 6 – Unsupervised clustering of MDL plots

**(A)** List of WGBS data used for the unsupervised clustering of MDL plots in Figure 4A. **(B and C)** Ward's clustering of the same WGBS data as (A) using the Spearman's rank-order correlation of the methylation levels of individual CpG sites (B) or sliding windows (window size, 1000 bp; step size, 1000 bp) (C).
