# Supplementary material for: Changepoint detection in base-resolution methylome data reveals a robust signature of methylated domain landscape
Source: BMC Genomics. 2015 Aug 12;16(1):594. doi: 10.1186/s12864-015-1809-5 (PMC4534107; doi:10.1186/s12864-015-1809-5)
Supplement: Additional file 7: — Effects of data size on MDL plots. (PDF 324 kb) [file 12864_2015_1809_MOESM7_ESM.pdf]

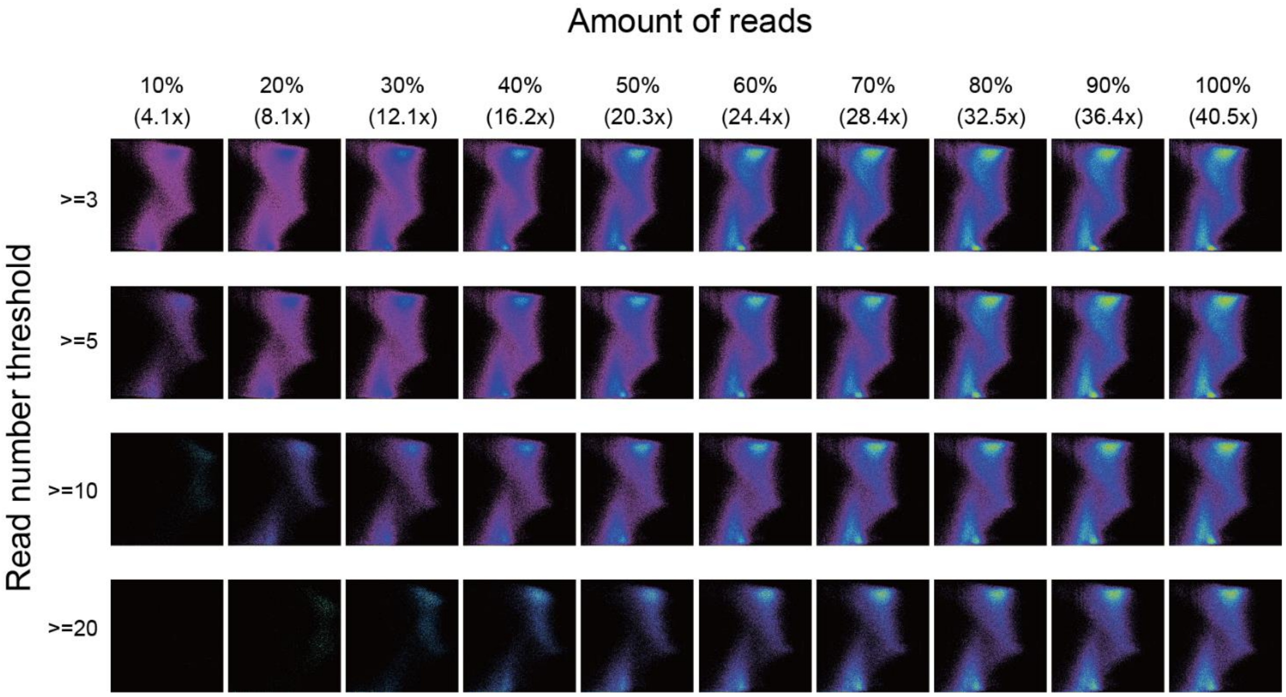

Additional file 7 – Effects of data size on MDL plots

The ten variously downsized IMR90 datasets were used for MDL plots under four different thresholds of minimal read depth (i.e., ≥3, ≥5, ≥10 or ≥20 reads). The number in parenthesis indicates the mean depth of genomic coverage.
