# Supplementary material for: Changepoint detection in base-resolution methylome data reveals a robust signature of methylated domain landscape
Source: BMC Genomics. 2015 Aug 12;16(1):594. doi: 10.1186/s12864-015-1809-5 (PMC4534107; doi:10.1186/s12864-015-1809-5)
Supplement: Additional file 8: — Performance comparison among methylome segmentation tools. (PDF 192 kb) [file 12864_2015_1809_MOESM8_ESM.pdf]

Tool	Major parameters	Computational time (hh:mm:ss)
SimpleChangepointCalculator	Penalty=1, Minimum Read Depth=5	00:10:49
R package “changepoint”	Penalty=default (AIC), Minimum Read Depth=5, Calculation of changepoint=PELT	01:01:06
R package “MethylSeekR”	Minimum Read Depth=10, Chromosome for training HMM for PMD masking=chr22	00:45:41

Additional file 8 – Performance comparison among methylome segmentation tools

Three tools for methylome segmentation were compared in terms of computational time using the IMR90 WGBS data [4] mapped to the human genome reference sequence hg18. Major parameters were set as indicated in the second column, whereas all the others were set as default. Computation was performed on a machine equipped with 24 CPU cores (Opteron 6168, 1.9 GHz) and a 64-GByte main memory. One CPU core or a single thread was assigned to each calculation and wall-clock time was measured.
